# Harmony or dissonance? The affordances of palliative care learning for emerging professional identity

**DOI:** 10.1007/s40037-020-00608-x

**Published:** 2020-08-27

**Authors:** Frances Kilbertus, Rola Ajjawi, Douglas Archibald

**Affiliations:** 1 Division of Clinical Science, Northern Ontario School of Medicine, Mindemoya, Ontario Canada; 2 Centre for Research in Assessment and Digital Learning, Deakin University, Melbourne, Australia; 3 Department of Family Medicine, University of Ottawa, Ottawa, Canada

**Keywords:** Palliative care learning, Professional identity, Personal narrative

## Abstract

**Introduction:**

Patient demographics demand physicians who are competent in and embrace palliative care as part of their professional identity. Published literature describes ways that learners acquire knowledge, skills and attitudes for palliative care. These studies are, however, limited by their focus on the individual where learning is about acquisition. Viewing learning as a process of becoming through the interplay of individual, social relationships and cultures, offers a novel perspective from which to explore the affordances for professional identity development.

**Methods:**

Qualitative narrative methods were used to explore 45 narratives of memorable learning (NMLs) for palliative care recounted by 14 graduating family medicine residents in one family medicine residency program. Thematic and narrative analyses identified the affordances that support and constrain the dynamic emergence of professional identity.

**Results:**

Participants recounted affordances that supported and/or constrained their learning acting on personal (e.g. past experiences of death), interpersonal (e.g. professional support) and systemic (e.g. patient continuity) levels. Opportunities for developing professional identity were dynamic: factors acted in harmony, were misaligned, or colliding to support or constrain an emerging professional identity for palliative care practice.

**Conclusion:**

Findings highlight how individual factors interplay with interpersonal and structural conditions in the workplace in dynamic and emergent ways that may support or constrain the emergence of professional identity. Viewing learning as a process of becoming allows teachers, curriculum developers and administrators to appreciate the complexity and importance of the interplay between the individual and the workplace affordances to create environments that nurture professional identity for palliative care practice.

## Introduction

Learning in health professions happens within complex and dynamic clinical workplaces [1, 2]. While the workplace context is acknowledged as an important factor in supporting learning and practice, attention to this influence and to the interplay of an individual within the workplace environment is lacking, particularly when using traditional research methods for educational interventions [3–5]. In this paper, we seek to explore palliative care learning using the metaphor of learning as becoming: a continuous, emergent process of interweaving individuals, social interactions and workplace cultures [6, 7]. The term affordances, borrowed from ecological psychology, describes what an environment provides for individuals, referring simultaneously to both the environment and the individual, and leads to possibilities for action [8]. An affordance is suggested by the social world and the degree to which individuals engage with them; “in other words, there is no guarantee that the ‘same’ invitation to participate is engaged with in the same way by different learners” [8, p. 117]. Therefore, an affordance in the social world may act to support some but constrain others. Through our empirical research we identify affordances for learning, using palliative care learning as specific case, thereby offering a broader perspective on the process of learning and professional identity development.

### Alternative perspectives of learning

Several metaphors have been used to describe how learning happens including: acquisition, participation and becoming [6, 9]. The acquisition metaphor, where individuals acquire knowledge and skills, is the most common metaphor for learning in medicine [10]. More recently, interest has been directed towards understanding the sociocultural influences on learning through workplace participation [2]. While neither metaphor is completely exclusive in its focus, each privileges a perspective: acquisition privileges individual agency, participation privileges social interactions and structures [9]. Conceptualizing learning as a process of becoming overcomes the limitation in the focus of these models by underlining that learning is, at all times, the intentional and unintentional interplay between individuals, social interactions and the culture of the learning environments [6]. This brings us to our earlier point that affordances are not taken up in the same way by different trainees. An important outcome of learning within this metaphor is the development of professional identity.

Along with acquisition of knowledge and skills, professional identity is increasingly recognized as a goal of medical education [10–13]. Different perspectives on identity have been presented ranging from singular, owned and stable; to dynamic, constructed and multiple [14]. For the purposes of this study we adopted the latter view, as developed by Jenkins: identification is a process not a thing, this process involves an individual, social interactions and cultural influences, and the individual is the character at the center of this complex process realizing him/herself in different ways in different contexts [14]. Jenkins asserts that “we should always be concerned with processes of identification, trajectories of being and becoming” (2014, p. 99). The way we think about learning can attune us to different features of this process, thus rendering previously unseen aspects of learning evident. In shifting the way we conceptualize how learning happens we can illuminate challenges that have not been considered and ask new questions as we work towards the goal of supporting learners’ emerging professional identity [15].

### Creating conditions to support learning and identity development

Helmich and colleagues proposed four paradigms that describe the interplay between emotion, meaning making and identity for medical students: insecurity, complying, developing and participating [16]. The paradigms of insecurity and complying, which did not support professional identity formation, were characterized as lacking engagement in meaningful participation, a focus on structural and procedural aspects of care and minimal opportunity for reflection. Developing and participating paradigms supported professional identity formation and were described as situations where: learners could actively explore the emotional dimensions of their work; there was developmental space for reflection to safely be curious, explorative and accept uncertainty; learners made active contributions to care and identified their learning needs. Our previous work highlights that these paradigms resonate with postgraduate learners as well as with medical students [7].

Embedded within the metaphor of learning as becoming is the broader notion that all experience contributes to learning, whether intended or unintended, thereby influencing emerging professional identity. From this perspective a question can be posed: what conditions support identity development for the desired outcome? Using the practice of palliative care as the case to explore this approach adds complexity. Palliative care requires a patient-centered holistic approach encompassing biomedical competence and compassionate caring. The domain of palliative care can be emotionally evocative and a cultural shift away from the disease-based, curative model that may challenge how learners see themselves as emerging practitioners [7, 17–26]. In most published literature on palliative care education, learning is considered as individualistic, following the acquisition metaphor. Reviews of palliative care learning have identified a myriad of ways that palliative care is taught in undergraduate and postgraduate medical education. Teaching and assessment of learning is conceptualised almost exclusively as learner acquisition, consistent with the dominant learning theory and traditional research methods [2, 27–30]. This perspective results either in minimizing relational and cultural issues in learning or viewing them as individual competencies with individual solutions [15, 31–33].

### Rationale and research questions

Demographics in many countries indicate a pressing need for physicians, generalist and specialist, who are competent in and embrace the practice of palliative care as part of their professional identity [26, 34, 35]. Deepening the understanding of how learners become physicians capable of providing compassionate palliative care is critical. Learning as a process of becoming that concurrently attunes to personal, interpersonal and systemic factors provides the framework to consider learning in a broader sense, with palliative care learning as a specific case, and to explore how affordances can support or constrain the emergence of professional identity for palliative care practice.

## Methods

Using a qualitative approach, we undertook a study of narratives of memorable learning (NMLs) for palliative care. Narrative methods, with a focus on the story, its underlying values and construction of meaning, are well suited to exploring the question of learning in complex environments and emerging professional identity [36–38]. Reflecting common usage, the terms narrative and story are used interchangeably. Volunteer participants were graduating family medicine residents from urban training sites affiliated with one Canadian family medicine residency program. Palliative care is a defined domain of care in a generalist competency framework for family medicine training in Canada [39, 40]. Details of the methodology and results of the broader thematic analysis from this study offering accounts of learning processes have been published elsewhere [7]. The current paper explores the interplay between personal factors, interpersonal influences and systemic conditions and how they support or constrain learning and the emergence of professional identity. The research ethics boards at the Ottawa Health Sciences Network (Protocol # 20140045-01H, April 23, 2014) and Bruyère Continuing Care (Protocol # M16-14-008, April 16, 2014) approved this study.

### Participant recruitment and sampling

Participants were recruited through a purposive sampling strategy [41]. An email invitation was sent by an administrative assistant to all 62 family medicine residents based at three urban training sites and who were preparing for their final certification exams during their last 6 months of training.

### Data collection and analysis

Participants were asked to recount stories of memorable learning in palliative care, aided by an interview guide following the structure of the personal incident narrative [25, 42]. All interviews were conducted by one interviewer known to participants but not in a position of power or authority (D.A.). Immersion in the data happened through listening to interview recordings and executing multiple close readings of each transcript. Three initial transcripts, representing one participant from each site, were reviewed by two researchers (F.K., R.A.), to identify discrete NMLs within each interview. The defining feature of a narrative that is key to its use in this study is the consequential linking of events to create meaning and give shape to things that might otherwise appear random [37, 38]. Each NML was analyzed to inductively develop themes and sub-themes for a coding framework [37, 38, 43]. The framework was discussed and refined iteratively to reach a shared understanding. The principal investigator (F.K.) undertook coding of all data using NVivo qualitative data analysis software version 10 (QSR International Pty Ltd., 2014). Coding was reviewed and discussed regularly with the team who brought diverse perspectives and roles to the analysis: insider (F.K., D.A.) and outsider (R.A.) views of the residency program, clinician teacher (F.K.) and education researchers (R.A., D.A.). Disagreements were resolved through discussion about theme and sub-theme definitions and the understanding of a contested quotation’s meaning. In order to deepen the understanding of the affordances of learning within individual NMLs, an in-depth narrative analysis was undertaken by R.A. and F.K. who interrogated and discussed each NML. Through close attention to *how* participants structured their stories, alongside *what* they said, the interplay between personal factors, interpersonal influences and systemic conditions were illuminated [37].

## Results

Interviews were conducted with 14 participants eliciting a total of 45 discrete NMLs that occurred in a variety of clinical and non-clinical settings [7]. Our analyses identified the personal, interpersonal and systemic affordances that supported or hindered learning within each NML. We acknowledge that there may be some overlap between these levels or they may be difficult to untangle. For simplicity, we will first present an overview of the affordances at different levels (Tab. 1), followed by exemplars from our participant narratives (Tab. 2). Then we set out longer vignettes to showcase the interplay between personal, interpersonal and systemic affordances within an NML and their influence on emerging professional identity [6].

**Table 1:** Levels of affordances in narratives of memorable learning

	Supports	Constraints
Personal factors	– Past involvement with death and dying: personal or professional – Actively defining learning needs – Seeking learning opportunities – Established reflective practice	– No exposure (or negative exposure) to palliative care during undergraduate medical education – Lack of experience with palliative care as a concept and/or clinical practice – Fatigue and burnout during residency
Interpersonal influences	– Professional support – Positive relationships: patients and families, mentors, role models – Positive non-professional relationships for support and reflection	– Lack of professional support – Communication challenges with patient and families: language, culture, family conflict, high negative emotional tone – Inadequate communication with interprofessional team about patient and family
Systemic conditions	– Well-structured and identified palliative care clinical learning opportunities – Time for communication – Continuity with patients and families – Formal curriculum: tutorials, lectures, books, online resources – Identification of and access to community-based resources	– Lack of identified and supported palliative care clinical learning opportunities – Lack of time for communication – Fragmentation of care – Obligatory referral of patients to specialized teams – Biomedical focus – Lack of access to interpreters

**Table 2:** Exemplars of factors supporting or constraining learning at personal, interpersonal and systemic levels

PERSONAL SUPPORTS AND CONSTRAINTS		
Support	Past experience with palliative care, death and dying	“… as a [clinical] clerk … we went to the palliative care unit and it was just very overwhelming … beyond the understanding of a med student at that time … but I think that might of shaped my view on palliative as a resident being more comfortable with these medications and everything” … P4
	Active engagement	“We had this blue book about palliative care and I just forced myself to read all the chapters basically to be more comfortable because I knew that it was one of the things I was not good in.” P9
Constraint	Lack of experience with palliative care, death and dying	“I studied medicine in [another country] … palliative care was non-existent at the time.” P14
	Challenge concept of palliative care	“… what I reflected on was that I still wanted to do active care like I still I wasn’t used to just focusing on goals of care … “ P1
INTERPERSONAL SUPPORTS AND CONSTRAINTS		
Support	Understanding social relationships within a community	“I knew her family … so it was a very intimate connection to the family. It was … a powerful thing for me …” P12
	Interprofessional team	“… we had meetings with nurses, with the pharmacist, with … the rest of the palliative care physicians … so definitely I never felt alone …” P13
Constraint	Language, cultural barrier and lack of team support	“… there was a huge cultural component … language component … and then trying to shoot off a little one liner sentence in morning rounds … when everyone really doesn’t have the time to listen …“ P10
	Uncertainty and negative emotions	“a big challenge is becoming involved in a situation where the family has expectations, the patient has expectations, there’s probably a lot of fear about what is going on and then … [I] show up without all the information and are expected to make a recommendation or a decision.” P5
SYSTEMIC SUPPORTS AND CONSTRAINTS		
Support	Well-structured clinical experience	“We do a family medicine palliative rotation … which is good because it shows you how you could fit palliative care into your family practice. You go to the hospice and then you come back and do your clinic and then you’re on call for the hospice so that’s good way to reflect what it might be like to do palliative care in the community later on.” P1
	Community supports	“… there’s a very good support system for family doctors with the [pain and symptom management team] …“ P13
Constraint	Lack of opportunity for holistic compassionate care	“… total pain … depression, … the spiritual aspect … it wasn’t addressed … we just didn’t have the opportunity to work with patients enough to … have that discussion …“ P10
	Multiple competing demands in the workplace	“For me it wasn’t too bad, but I have heard for other residents … they didn’t always have their patients blocked in the afternoon … they’d have a full patient roster scheduled in the afternoon and then they’d have a hospice admission and they’d be like ‘Oh well someone forgot to schedule my patients off and I’ve got to see patients ’till 5:30, then go to [hospice] and maybe do an admission and then I’ve got to go home to my family.’” P11

Personal factors included past experience, dispositions, knowledge, skill, beliefs and emotions. Interpersonal influences included relationships and interactions co-created with others. Systemic conditions included the structures of healthcare and educational institutions and workplaces wherein memorable learning happened. Within discrete NMLs, elements supporting or hindering learning were present in ways that aligned along personal, interpersonal and systemic levels to fully support learning as becoming or collided resulting in confusion or mixed messages.

### Personal factors

Past involvement with death and dying enabled palliative care learning: in formal medical education, in previous domains of study or careers and in personal life experience. The consequences of these experiences for participants were described as increased understanding of the concept of palliative care, more comfort and confidence to participate in care of the dying and a sense of personal meaning derived from the work. An individual disposition towards actively engaging in defining learning needs, seeking learning opportunities and accessing both human (the interprofessional team) and formal curricular (books, tutorials, online tools) resources for palliative care supported learning. In contrast, lack of or negative past experience with palliative care as a concept and a practice were identified as personal barriers to learning. More globally, feeling fatigued and burnt-out during residency training interfered with learning.

### Interpersonal influences

Positive relationships with colleagues, interprofessional team members, supervising physicians, patients and families were frequently identified as factors enabling learning. This supported effective communication, relationship building and a holistic approach to care. Having positive mentors, role models and supervisors, and observing their participation in the workplace supported learning and contributed to creating positive learning environments. Positive relationships outside of the workplace, such as close friends and family members with whom participants could reflect upon their work and its personal repercussions, were identified as strategies that supported learning. Lack of perceived support from supervisors, colleagues or other team members interfered with learning. Reasons for this included lack of time and interest by team members, a focus on acute curative care and differing points of view that were not resolved. Communication challenges were described as a result of language and cultural barriers, uncertainty about a patient’s history and disease trajectory, difficult family dynamics and high emotional tone.

### Systemic conditions

Systemic enablers to learning were described as having well-structured and well-scheduled opportunities to participate in clinical palliative care, both core and elective, in hospitals, residential hospices, outpatient clinics and homes. Resources that enabled these clinical experiences were: time to spend with patients and families; formal curricular elements such as books, tutorials and on-line tools; access to community-based interprofessional teams; and access to in-hospital specialist palliative care teams. Having continuity with patients and families along the illness trajectory supported learning. Conversely, lack of identified palliative care opportunities in primary care practices, specialist rotations or in specialized palliative care units hindered learning. Lack of time to spend with patients and families, particularly around unexpected or catastrophic changes, and multiple competing demands due to the nature of clinical rotations and scheduling interfered with learning. Several participants remarked on the absence of any home-based care during their medical education and how this was a barrier to learning to practice community-based palliative care. Within some tertiary care settings, fragmentation of care or the requirement to transfer patient care to a specialized palliative care team with subsequent loss of continuity was identified as a barrier to learning.

### Interplay of factors at multiple levels and the emergence of professional identity

We illustrate three vignettes that highlight how factors at multiple level align, misalign or collide within an NML influencing professional identity. Participant 7 (Infobox 1), an individual with prior experiences of death and dying, described taking up affordances within the environment such as actively engaging in defining learning needs, seeking learning opportunities, and accessing both human (the interprofessional team) and formal curricular resources (books, tutorials, online tools) for palliative care. In contrast, for Participant 5, lack of past experience with palliative care as a concept and a practice were identified as personal barriers interfering with learning. In other NMLs this resulted in lack of confidence and feeling overwhelmed and uncertain about being able to engage in this practice in the future. For Participant 5, this personal barrier was offset by interpersonal and systemic conditions supporting learning (Infobox 2). Confusion resulted when an individual with positive personal experiences of death and dying confronted interpersonal and/or systemic challenges to participating in this care that were overwhelming (Infobox 3).

### Vignette 1: Alignment personal, interpersonal, systemic levels: harmony

Participant 7 described her familiarity with palliative care, recalling the physician who cared for her grandfather and describing previous palliative care learning (formal and workplace). These personal and systemic factors supported her emerging identity for palliative care practice. She further describes multiple positive interpersonal factors supporting her within the NML: interactions with interprofessional colleagues and family members. She was afforded and seized the opportunity for meaningful engagement. This enabled her to deal with uncertainty and emotions and opened a reflective space. She narrates her story as an active care provider for this dying man and his family.

#### Box 1 Vignette 1

“… [Palliative care nurse] gives some good lectures… She does a lot of good teaching… The other way [I learned about palliative care] was definitely the hospice experience … I was on the [hospital] service, [and there was] an elderly guy … he wasn’t doing well at home, and then all of the sudden I got called that he was having trouble breathing … he got drowsier and drowsier and he was starting to fatigue and I called his family and told them to come in and then had to kind of on the fly … try to remember what kind of medications you give cause he wasn’t going to continue this way for very long. … I called palliative care on call just to ask them … what medications they would give or what doses … to just make sure he was comfortable … The family didn’t make it in time. They came in and he had already passed … We had a really good conversation with them afterwards … we all just talked about the patient and his life and what had happened … it had been quite unexpected … I think so much of what they’ll remember about [that] night … is going to be the conversation that they had with me and the nurse … everyone should have the right to die with dignity … [I was able] to actually do something pretty nice for someone. Like I still remember the doctor who took care of my grandfather when he was passing away.” Participant 7

… *denotes text edited for brevity*

### Vignette 2: Misalignment personal, interpersonal, systemic levels: satisfaction

Participant 5 had no experience with palliative care during his undergraduate medical education and although he heard about this practice through classmates, he was uncertain about what it was and what it entailed. Thus, he arrived at his first clinical palliative care experience as a postgraduate trainee. Despite his uncertainty and lack of confidence, he was curious and self-directed and was able to establish relationships with patient, family and team members to fully participate in this care. The structure of the workplace and schedule allowed him time to overcome these barriers, engage his curiosity, deal with uncertainty, and fully participate in the care of this man and his family. This resulted in memorable learning, satisfaction, and his presentation as someone capable of engaging in this care.

#### Box 2 Vignette 2

“In med school I had no palliative care exposure. It was a selective that I didn’t know anything about, and I didn’t really get exposed to it … I was called on a weekend in first year residency … to do an admission to [the hospice] and I remember thinking, … this is not good because I don’t even know where to start … so I was doing some reading and I was talking to the family and then when I talked to my staff it became apparent that I didn’t even really have an approach to palliative care. … and [the attending physician] was great. We spent probably half hour on the phone talking about everything that I needed to make sure I covered … he was so clear that like he wanted me to feel independent in dealing with this and would definitely come in if I felt it was necessary … I really liked that independence and I liked the guidance that he gave me … I spent several hours … coming up with the plan, talking with the nurses, making sure the orders were correct and making sure that there was a plan in place … I felt proud of myself that I had made it through that experience and had a good rapport with the patient and was able to call [the attending physician] and talk about my plan, and tweak it a little bit and felt like … I ’ve definitely learned something here today.” Participant 5

### Vignette 3: Collision of personal, interpersonal, systemic levels: dissonance

Participant 13 described herself as someone who is familiar with palliative care from several perspectives: family, past jobs and her previous health profession. Her NML is a dramatic story of challenging interpersonal interactions with a family in crisis, exacerbated by systemic factors of multiple responsibilities while on duty. Despite her personal disposition and awareness of palliative care practice, the need to comply with the pervasive systemic challenges and resulting unresolved emotions caused frustration. She described feeling conflicted and compromised about how she aspired to be and to act in this interaction, asking, ‘how is this ok?’. The sociocultural factors in this NML collide with her individual dispositions. While she presents herself as a caring and compassionate person, the conditions under which she was working did not allow her to express nor resolve this. The result is confusion about who she is and how she acted in this circumstance.

#### Box 3 Vignette 3

“… Before medicine I [was a heath professional] … in that practice … I was exposed to palliative care … [a family member] used to be a palliative care nurse … I remember her speaking about it. I used to be a [previous job] on the oncology floor … so it’s been around me … I received [a call] to go down to the emergency department to see a woman who had just been told that she had metastatic disease and that she … was full of cancer all over her body and I was to go share this with her … it was like two o ’clock in the morning and I was in charge of like ten women who were laboring and I go downstairs to the [emergency department]. All of her kids are around her, and I had to share this with her and then my pager went off. And all of the kids had different reactions. Some were sobbing, some were crying, some were yelling at me … and I was really trying hard to communicate … and just to … say … ‘I really don’t have much time.’ … I just felt very frustrated because I got called [away]. Then I had to leave. And I felt like that family was just left … in the dust and … and I felt as though, ‘How is that okay?’ … I was never allowed really … well I was never … able to go back and to see that family in the emergency department again … because it was just so crazy … so I felt quite conflicted inside …”. Participant 13

## Discussion

Over 25 years ago, when considering palliative care education, James and MacLeod stated, “Prior experience is likely to shape both the way professionals learn and their learning needs, their perspectives of palliative care, what they consider to be important priorities, how they relate to others involved in the care process, and their ability to articulate and explain the bases of their actions.” [17, p. 8]. The learner, with his/her unique life experiences, enters the complex and dynamic world of clinical palliative care and both are shaped through the process. This space defines the “horizon of learning”: how the individual, social interactions and workplace structures interact in ways that support or hinder the process of identity formation [6, p. 43].

Following Helmich and colleagues’ paradigms of developing and participating that support professional identity formation, individual factors may interplay with interpersonal influences and systemic conditions in ways that support the emergence of professional identity; they may be misaligned but support professional identity for the desired outcome nonetheless through opening of spaces to engage, to participate, to feel and to explore [16]. There may be deep dissonance, resulting in mixed messages and confusion between individual aspirations and what is demanded of a learner in the workplace, resulting in the paradigms of insecurity and complying [16]. In the absence of reflection and exploration of these interpersonal and systemic affordances and their interplay with an individual, the influence on emerging identity is unknown and possibly contrary to the desired educational outcomes.

How we conceptualize learning matters. This study highlights the inherent limitation in a singular conceptualization of learning. Participants in this study recounted NMLs describing personal characteristics, orientations and dispositions that they brought to the complex clinical workplace. They then interacted with the affordances of the social and cultural milieu of the workplace resulting in experiences that supported, hindered or confused the process of becoming a physician with a professional identity for palliative care practice. All these experiences, both positive and negative, easy and difficult, were recounted as memorable learning. Illuminating, exploring, and valuing the interplay of factors at multiple levels is necessary to understand the broader experience of becoming a physician with an emerging professional identity for palliative care practice. Furthermore, broadening the conceptualization of learning can illuminate inconsistent messages in the clinical workplace. For example, although formal educational and institutional rhetoric may favor patient-centered values, this may not be consistent with the lived experiences of learners within these institutions [15, 22, 44]. By making these gaps visible, an opportunity is afforded to explore implications for emerging professional identity; to address challenges at the appropriate level; and to align institutional values, lived experience of learners, and the desired outcomes of education.

### Implications

Viewing learning as a becoming allows teachers, clinicians and educators, through story, to: attune to the multiple constructions of learner identity, attend to and embrace uncertainty in learners, allow and explore the emotions experienced through learning in complex clinical contexts, and consider how the interplay of the individual and their relationships within the culture of the learning environment supports or hinders emerging professional identity. Situations that confuse or present mixed messages to learners can be identified and addressed at the appropriate level. Opening a space for reflection and reflexive engagement and valuing this as learning will contribute to the process of becoming physicians.

### Limitations

This study was limited by having participants from urban sites within one residency program. The non-urban perspective was not included. The voices of those who may have had negative or traumatic experiences or who did not identify with this area of practice may not have been heard and their absence may have illuminated different patterns of intersection. This study considered the case for palliative care learning. Further study will be required to consider this approach in other clinical learning domains.

## Conclusion

Conceptualizing learning as a process of becoming that is inclusive of professional identity enables teachers, clinicians, curriculum developers and administrators to understand, explore and act at multiple levels to align values and goals with the lived experience of learners and the desired educational outcome.
